# Pelvis-Based Three-Dimensional Hip Morphology in Japanese Women: Reference Distributions and Spatial Relationships of Femoral Head Position, Acetabular Orientation, and Coverage

**DOI:** 10.7759/cureus.117680

**Published:** 2026-10-09

**Authors:** Nozomi Murakami, Ryoichi Izumida, Shojiro Katoh, Osamu Hemmi, Hiroyasu Ikegami, Osahiko Tsuji

**Affiliations:** 1 Department of Orthopedic Surgery, Toho University Ohashi Medical Center, Tokyo, JPN; 2 Department of Orthopedic Surgery, Edogawa Hospital, Tokyo, JPN; 3 Keiyu Artificial Joint Center, Edogawa Hospital, Tokyo, JPN

**Keywords:** acetabular orientation, femoral head coverage, femoral head position, hip morphology, three-dimensional computed tomography

## Abstract

Background: Conventional radiographic assessment of the hip primarily describes coronal acetabular coverage and does not fully characterize the three-dimensional spatial relationship among the pelvis, femoral head, and acetabulum. This study aimed to establish a pelvis-based three-dimensional morphometric reference framework for femoral head position, acetabular orientation, and regional coverage in Japanese women without clinically recognized hip disease.

Methods: This retrospective cross-sectional study included 172 Japanese women (344 hips; mean age, 45.8 ± 10.7 years; range, 19-59 years) who underwent chest-abdominal computed tomography for nonorthopedic indications. Three-dimensional reconstructions were standardized using the anatomic pelvic plane. Femoral head position was quantified using the anterior index (AI) and lateralization index (LI), and acetabular morphology was assessed using acetabular anteversion (AV), anterior, posterior, and horizontal acetabular sector angles, conventional coverage parameters, and coverage areas. Statistical analyses accounted for clustering of bilateral hips. Sensitivity analyses were performed in hips with a center-edge angle of 25°-40° and in right hips only.

Results: The mean AI and LI were 62.5 ± 3.3 and 68.4 ± 5.7, respectively. After adjustment for age, side, and bilateral clustering, greater AI was associated with greater AV (standardized β = 0.62), smaller anterior acetabular sector angle (β = -0.40), and larger posterior acetabular sector angle (β = 0.32; all p < 0.001). LI showed weaker associations with acetabular orientation and regional coverage. No statistically significant side-to-side or age-related differences were observed in the principal three-dimensional positional and angular parameters. The principal associations were preserved in both sensitivity analyses.

Conclusions: Pelvis-relative femoral head position was consistently associated with acetabular orientation and the anterior-posterior distribution of regional coverage. These findings provide a population-specific three-dimensional anatomical reference framework that complements conventional coronal hip measurements and may serve as a basis for future comparisons with morphologically abnormal hips.

## Introduction

Hip morphology has traditionally been evaluated primarily using anteroposterior radiographs, with parameters such as the center-edge (CE) angle and Sharp angle providing clinically useful information on coronal acetabular morphology and coverage. However, these two-dimensional measurements cannot fully characterize the three-dimensional spatial relationship among the pelvis, acetabulum, and femoral head. Computed tomography (CT) enables assessment of femoral head position, acetabular orientation, and regional coverage within a three-dimensional pelvic coordinate system. An early CT-based analysis of Japanese adults quantified the position of the femoral head within the pelvis using anterior and lateral indices together with acetabular anteversion (AV) and anterior acetabular sector angle (AASA), posterior acetabular sector angle (PASA), and horizontal acetabular sector angle (HASA), suggesting that hip morphology can be characterized by a set of spatial relationships between the femoral head and pelvis [1]. Subsequent CT studies of asymptomatic populations have demonstrated substantial variation in acetabular coverage and orientation that may not be fully represented by conventional radiographic thresholds [2]. Large three-dimensional CT studies have further shown that reference ranges for hip morphology may vary according to sex, age, and other individual characteristics, emphasizing the need to define reference morphology within well-characterized populations [3]. Similar sex- and age-related variation in acetabular measurements has also been demonstrated in Japanese adults [4]. Collectively, these observations support characterization of the native hip as a three-dimensional spatial relationship incorporating femoral head position, acetabular orientation, and femoral head coverage relative to a standardized pelvic reference system.

Evidence from pathologic hips further suggests that the position of the hip center relative to the pelvis represents a distinct component of three-dimensional hip morphology. In CT analyses of secondary osteoarthritis associated with acetabular dysplasia, Ishibashi et al. reported that the hip center was positioned more anterolaterally than in reference hips, with increasing lateralization and decreasing acetabular AV as osteoarthritis progressed [5]. Henmi subsequently extended this approach using three-dimensional CT and demonstrated that secondary coxarthrosis was characterized not only by lateral malposition but also by anterior malposition of the femoral head within the pelvis, together with regional differences in acetabular coverage [6]. More recently, an anterior-pelvic-plane-based analysis independently demonstrated that femoral head centers in developmental dysplasia were displaced anteriorly and laterally relative to healthy controls, supporting the relevance of pelvic-coordinate-based assessment of femoral head position [7]. Contemporary three-dimensional studies have also demonstrated that acetabular dysplasia is characterized by heterogeneous regional deficiencies in femoral head coverage that cannot be fully represented by a single coronal measurement [8].

Despite these advances, the reference spatial relationship between the femoral head and pelvis remains incompletely characterized. An exploratory CT study quantified three-dimensional hip-center coordinates relative to pelvic dimensions, but included only 37 hips, most derived from the contralateral side of patients evaluated for unilateral hip pain or acetabular fracture [9]. Moreover, larger CT-based reference studies have primarily focused on acetabular orientation and coverage, and some have defined the reference cohort by excluding hips outside predefined two-dimensional coverage thresholds before deriving three-dimensional reference values [2]. Such approaches may limit the ability to characterize the natural distribution of three-dimensional hip morphology and may introduce circularity when conventional coverage parameters are used to define the reference population. These limitations support the need for a well-characterized reference distribution that integrates pelvic-relative femoral head position with acetabular orientation and regional coverage without defining the study population solely on the basis of preselected coronal coverage criteria. Because pelvic and acetabular morphology may vary by sex [3,4], the present cohort was restricted to women to establish a sex-specific reference distribution rather than combining male and female morphology within a single reference framework.

Accordingly, this study aimed to characterize the distribution and spatial relationships of pelvis-relative femoral head position, acetabular orientation, and regional projection-based femoral head coverage within a standardized three-dimensional pelvic coordinate system in Japanese women without clinically recognized hip disease or overt degenerative or destructive abnormalities. Femoral head position was evaluated using the anterior index (AI) and lateralization index (LI); acetabular orientation and regional angular coverage were evaluated using AV, AASA, PASA, and HASA; and projection-based overall, anterior, and posterior coverage areas were quantified in square millimeters. An anatomic pelvic plane (APP)-standardized coordinate system was used to distinguish intrinsic pelvis-relative morphology from variation attributable to pelvic orientation during CT acquisition and to complement conventional coronal radiographic measurements. We hypothesized that pelvis-relative femoral head position would be systematically associated with acetabular orientation and regional coverage, whereas the principal positional and angular parameters would show limited variation according to side and age within the observed adult age range.

## Materials and methods

Study design, setting, and ethics

This retrospective observational cross-sectional morphometric study was conducted at Edogawa Hospital using existing chest-abdominal CT examinations obtained for nonorthopedic indications between May 1, 2023, and May 31, 2024. The study protocol was approved by the Ethics Committee and Institutional Review Board of Edogawa Hospital on April 28, 2023 (Approval No. 12). All procedures were conducted in accordance with the Declaration of Helsinki and its subsequent amendments. Because the study used retrospectively collected imaging data, relevant study information was publicly disclosed, and participants were provided with the opportunity to opt out of the study.

Study population and eligibility criteria

During the study period, all eligible consecutive women younger than 60 years who underwent chest-abdominal CT for nonorthopedic indications, including health screening and other medical evaluations unrelated to the hip, were screened for inclusion. Clinical records and CT images were reviewed to confirm the absence of clinically recognized hip disease and overt degenerative or destructive abnormalities. Screening also included assessment for prior hip or pelvic surgery, hip or pelvic fracture, implants, and metal artifacts that could interfere with morphometric measurement. A total of 172 women (344 hips), aged 19-59 years, met these criteria and were included in the study. Both hips of each participant were included in the analysis.

The CE angle was not used as an eligibility criterion because lateral acetabular coverage was one of the morphologic characteristics evaluated in this study. Instead, the CE angle was retained as an analytical variable. A subgroup with conventionally normal lateral acetabular coverage, defined as a CT-based CE angle of 25°-40°, was evaluated in a sensitivity analysis.

CT acquisition and three-dimensional reconstruction

CT examinations were performed using Discovery CT750HD, Revolution CT, or Revolution EVO scanners (GE Healthcare, Chicago, IL) under routine clinical acquisition protocols. Images were reconstructed with a slice thickness of 0.625 mm. For image review and three-dimensional reconstruction, a window width of 350 Hounsfield units and a window level of 50 Hounsfield units were used.

All examinations were obtained with the participants in the supine position. The imaging field extended from the lower lumbar region through the proximal femur, allowing visualization of the entire pelvic ring and both hip joints. Three-dimensional reconstructions were generated from the original CT datasets using AW Volume Share 5 (GE Healthcare) and Zio Station 2+ (Ziosoft, Tokyo, Japan).

The reconstructed images were subsequently standardized to a pelvis-based anatomical coordinate system before morphometric measurements were performed.

Pelvic coordinate system

A standardized pelvis-based coordinate system was established before morphometric measurements. The APP was defined using three anatomical landmarks: the bilateral anterior superior iliac spines (ASISs) and the midline point of the anterior surface of the pubic symphysis.

Three-dimensional pelvic reconstructions were reoriented to the APP-based reference system. First, on the inferior view, the pelvis was rotated until the anterior borders of the right and left ilia were aligned with the reference grid (Figure 1A). The pelvis was then viewed laterally and further rotated until the iliac contour and pubic symphysis were vertically aligned with the reference grid (Figure 1B). After rotation back to the frontal view, the standardized pelvic orientation was established with the APP facing anteriorly (Figure 2).

**Figure 1:**
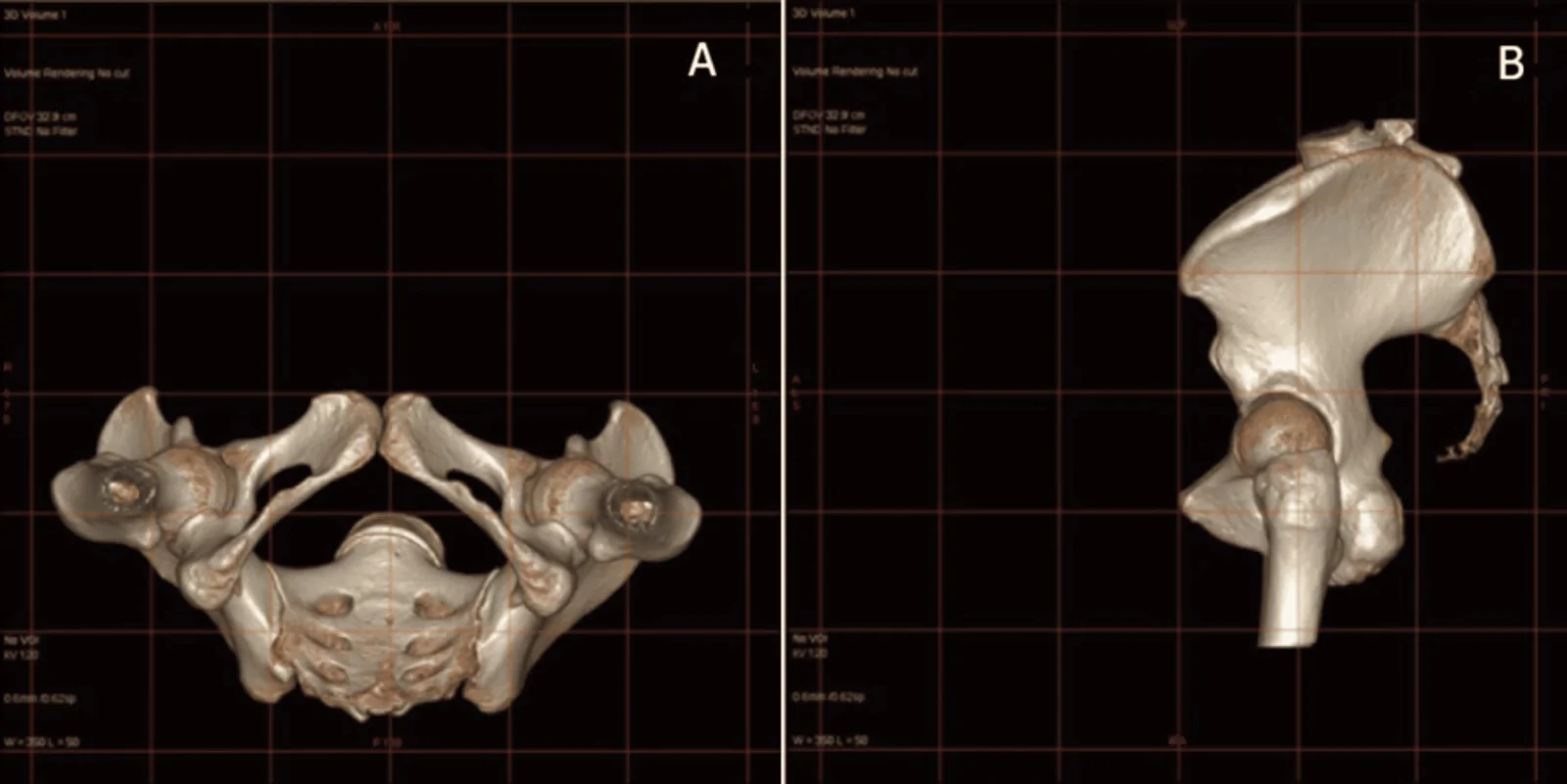
Three-dimensional coordinate transformation and pelvic standardization Computed tomography images were acquired with the participants in the supine position, and three-dimensional reconstructions were generated from abdominal-pelvic computed tomography data extending from the lower lumbar spine to the proximal femur. (A) Inferior view of the pelvis during standardization to the anatomic pelvic plane. The pelvis was rotated until the anterior borders of the right and left ilia were aligned with the reference grid. (B) Lateral view of the pelvis after reorientation. The pelvis was rotated until the iliac contour and pubic symphysis were vertically aligned with the reference grid, establishing the standardized pelvic orientation used for subsequent morphometric measurements

**Figure 2:**
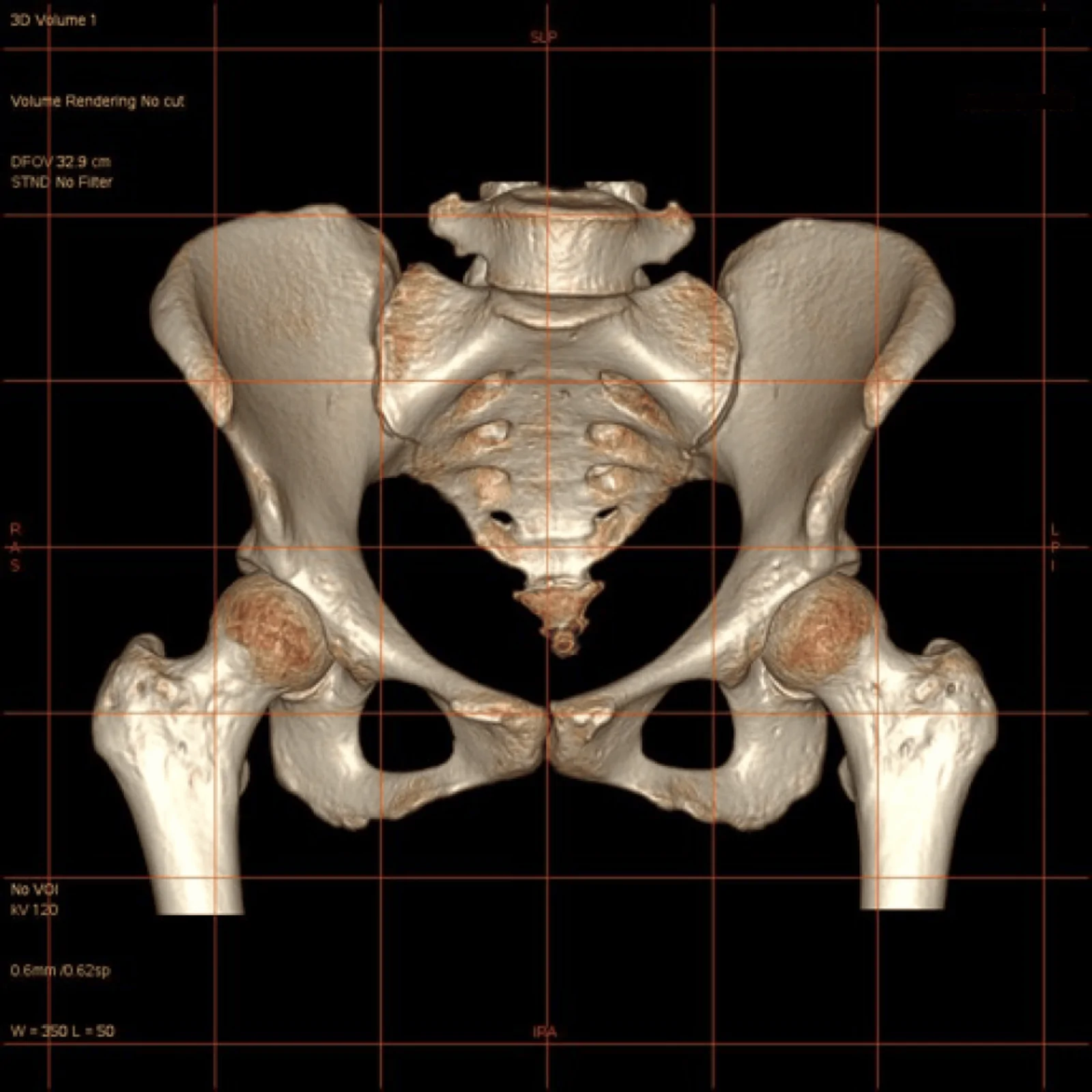
Standardized pelvic orientation based on the anatomic pelvic plane Frontal view of the three-dimensional pelvic reconstruction after the coordinate transformation shown in Figure 1. Following alignment in the inferior and lateral views, the pelvis was returned to the frontal view, establishing the standardized anatomic pelvic plane-based coordinate system used for subsequent morphometric measurements

This APP-based coordinate system served as the anatomical reference for all subsequent measurements. It was used to standardize anatomical orientation across participants and was not intended to represent functional standing pelvic alignment.

Determination of the femoral head center

The center of each femoral head was determined using a cross-grid method on the standardized three-dimensional CT reconstruction. After reducing or removing the pelvic rendering, the femoral head was displayed in isolation. A cross-shaped grid was adjusted to encompass the femoral head, with the grid width fitted to the maximal extent of the femoral head on the frontal view (Figure 3A). The reconstruction was then rotated by 90° to confirm that the femoral head remained enclosed by the same grid on the superior view (Figure 3B).

**Figure 3:**
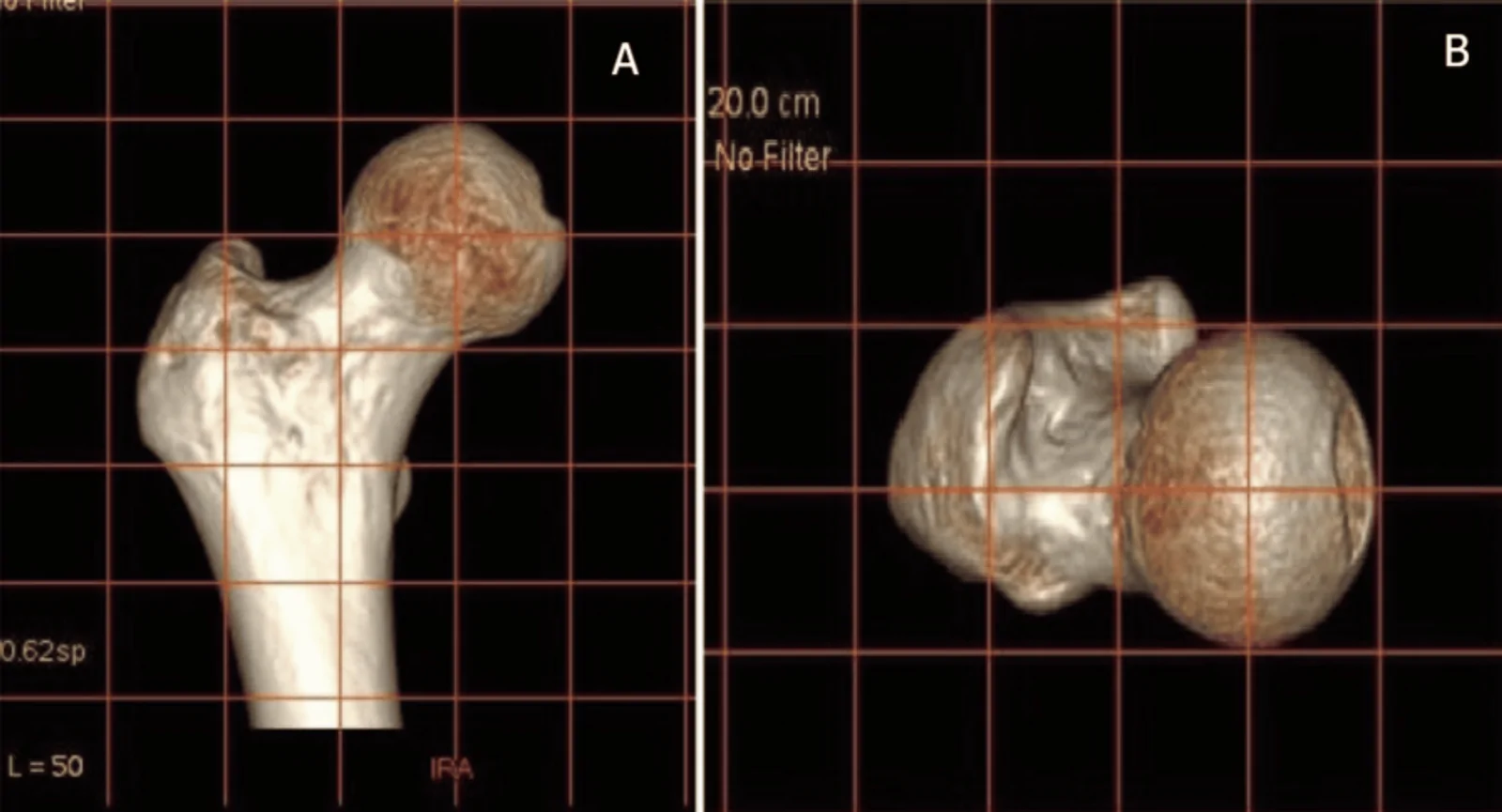
Determination of the femoral head center using the cross-grid method (A) Frontal view of the three-dimensional femoral head reconstruction. A cross-shaped reference grid was adjusted to encompass the femoral head. (B) Superior view obtained after rotating the reconstruction by 90°, confirming that the femoral head remained enclosed within the same grid. This procedure was used to identify the three-dimensional center of the femoral head

The intersection of the orthogonal grid lines was identified on the frontal view (Figure 4A) and confirmed at the same position on the superior view (Figure 4B). The verified intersection point was considered the three-dimensional center of the femoral head. Because a marker placed directly on the three-dimensional surface would be assigned to the surface rather than the internal center, the final center point was identified on the corresponding sectional CT images. A provisional marker was first placed on the three-dimensional reconstruction, and the axial slices were advanced while monitoring the linked marker position. The slice corresponding to the cross-grid intersection was then identified, and the definitive femoral head center was marked on the sectional image. The same procedure was repeated for the contralateral hip.

**Figure 4:**
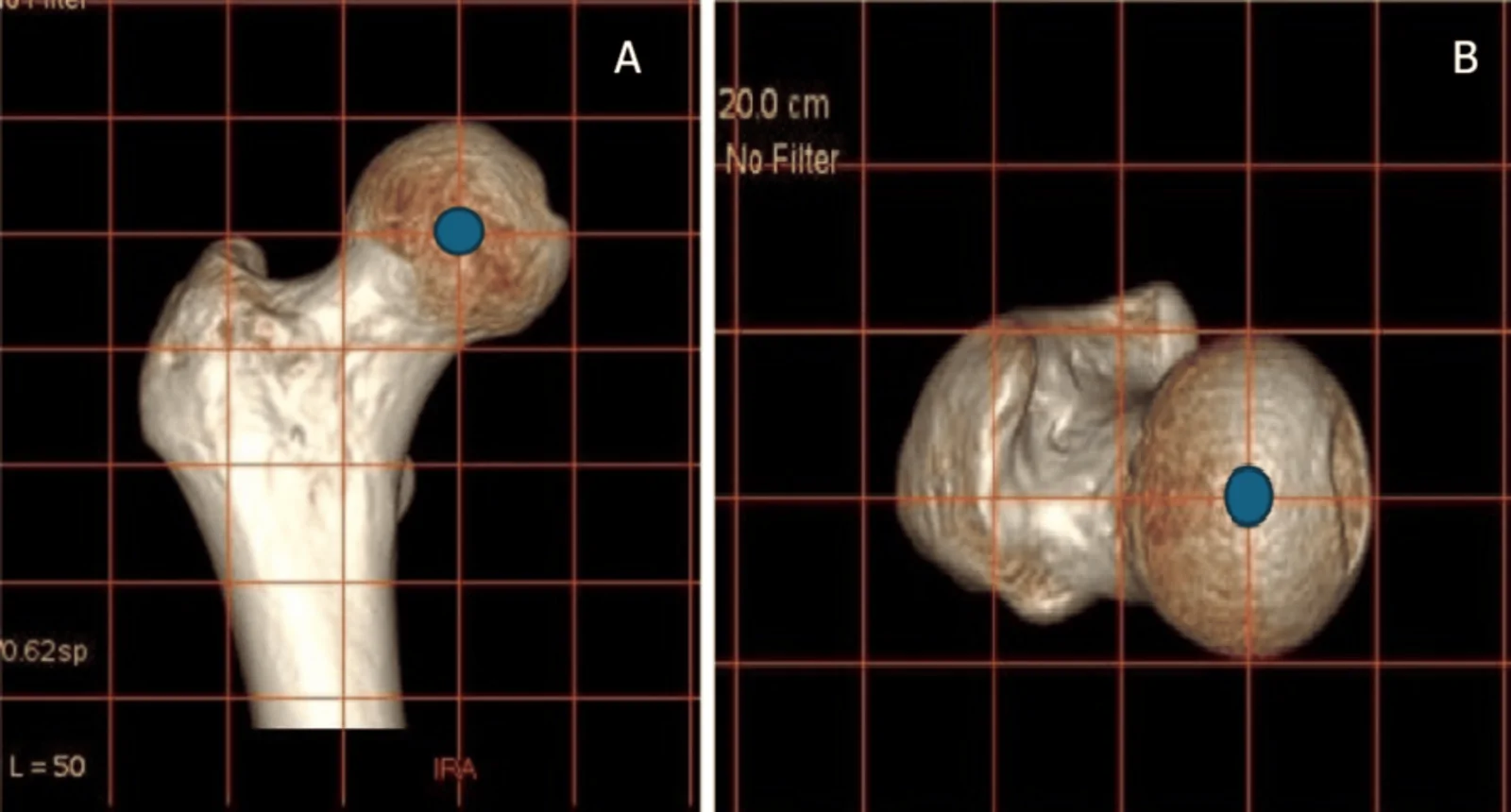
Identification of the femoral head center (A) Frontal view of the three-dimensional femoral head reconstruction showing the intersection of the orthogonal grid lines, indicated by the blue marker. (B) Superior view confirming the same intersection point after rotation of the reconstruction. The verified intersection point was defined as the three-dimensional center of the femoral head and was used as the reference point for subsequent morphometric measurements

The bilateral femoral head centers identified in this manner were used as reference points for subsequent measurements of femoral head position, acetabular orientation, sector angles, and coverage area. All femoral head centers were determined manually using this cross-grid procedure. No automated sphere-fitting, least-squares sphere-fitting, or volumetric centroid algorithm was used.

Femoral head position

The position of the femoral head center within the pelvis was quantified using the LI and AI. The femoral head center identified on the standardized three-dimensional CT reconstruction was treated as a point in three-dimensional space, and its mediolateral and anteroposterior positional components were used to calculate the LI and AI, respectively. The femoral head center was not transferred to the axial level of the pelvic landmarks, and no separate axial-plane reprojection was performed.

The LI quantified the mediolateral position of the femoral head center relative to the pelvis. The mediolateral distance from the midsagittal plane to the femoral head center was normalized by one-half of the maximum transverse pelvic diameter at the level of the ASISs (Figure 5). The LI was calculated as follows:

\(\mathrm{LI} = \left[\frac{\text{Distance from the midsagittal plane to the femoral head center}} {\text{Maximum transverse pelvic diameter}/2} \right] \times 100\).

**Figure 5:**
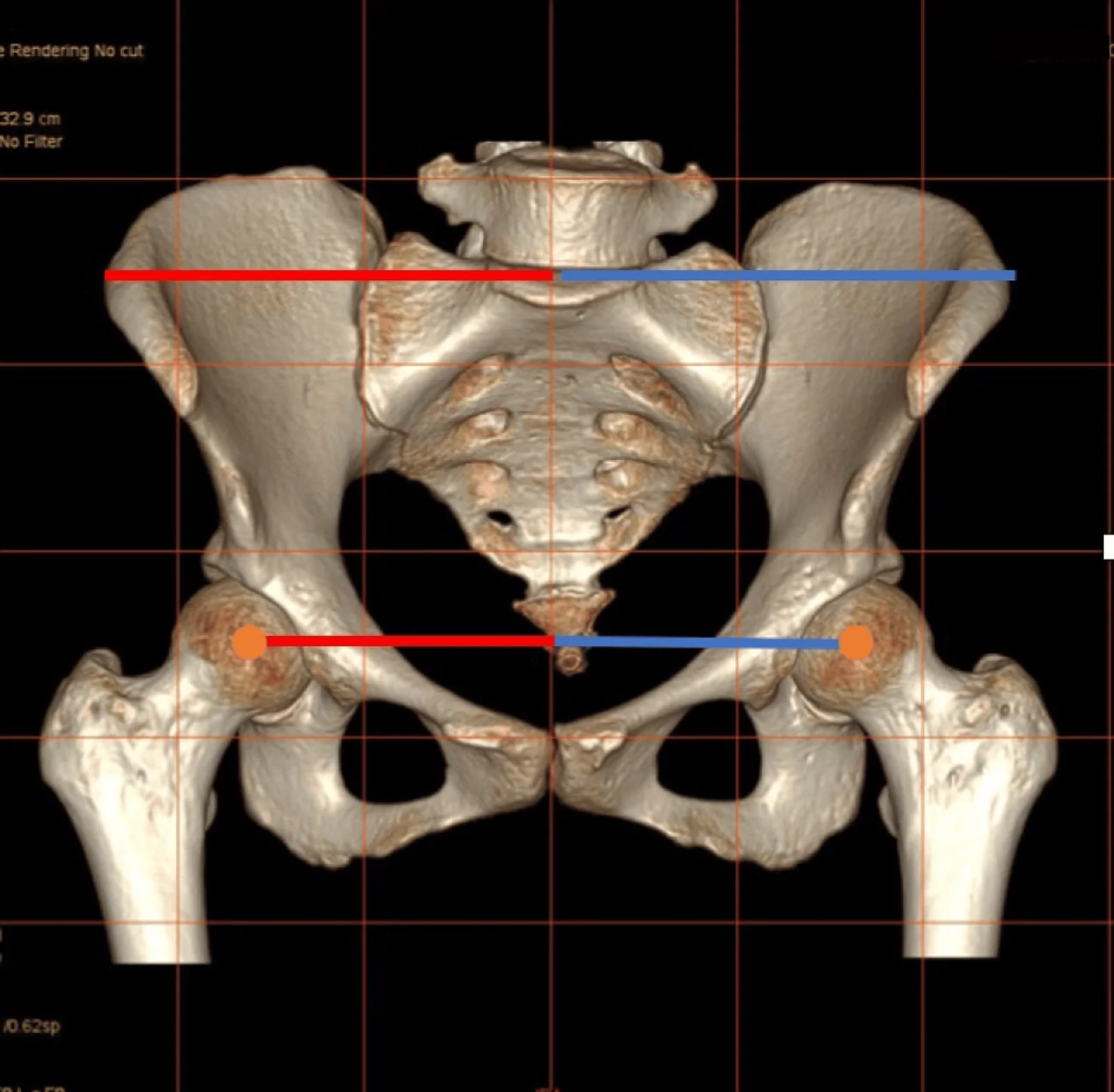
Measurement of the lateralization index of the femoral head The lateralization index was calculated from the mediolateral component of the three-dimensional femoral head center as the distance from the midsagittal plane to the femoral head center divided by one-half of the maximum transverse pelvic diameter and multiplied by 100. The femoral head center was not reprojected to a separate axial level for this measurement

The AI quantified the anteroposterior position of the femoral head center relative to the pelvis. The posterior superior iliac spine (PSIS) and ASIS were used as the posterior and anterior reference positions, respectively. The PSIS was assigned a value of 0 and the ASIS a value of 100. The anteroposterior positional component of the femoral head center was expressed relative to this PSIS-to-ASIS reference span as a percentage (Figure 6). The AI was calculated as follows:

\(\mathrm{AI} = \left[ \frac{\text{Anteroposterior distance of the femoral head center from the PSIS reference position}} {\text{PSIS-to-ASIS anteroposterior reference distance}} \right] \times 100\).

**Figure 6:**
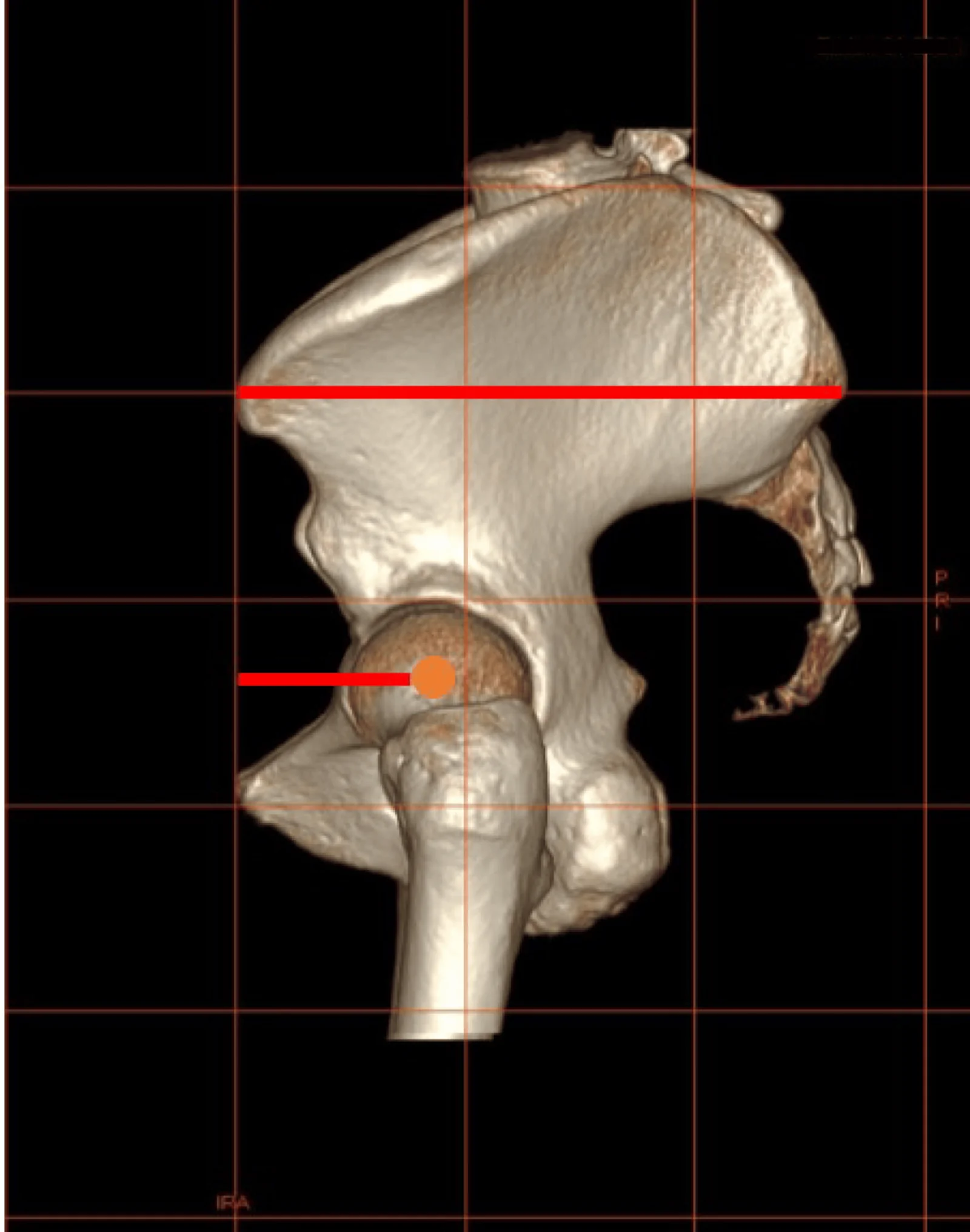
Measurement of the anterior index of the femoral head The anterior index was calculated from the anteroposterior component of the three-dimensional femoral head center relative to the PSIS-to-ASIS reference distance. The PSIS was assigned a value of 0 and the ASIS a value of 100, and the femoral head center position was expressed as a percentage of this anteroposterior reference span. The femoral head center was not transferred to the axial level of the ASIS or PSIS PSIS: posterior superior iliac spine; ASIS: anterior superior iliac spine

Thus, the LI and AI represented normalized mediolateral and anteroposterior components, respectively, of the three-dimensional femoral head center relative to pelvis-based reference dimensions, rather than measurements obtained after relocating the femoral head center to the level of the ASIS or PSIS.

Acetabular orientation

Acetabular orientation was quantified using the AV angle. On the axial plane passing through the center of the femoral head, AV was defined as the angle between the line connecting the anterior and posterior acetabular rims and the sagittal plane of the standardized pelvic coordinate system (Figure 7).

**Figure 7:**
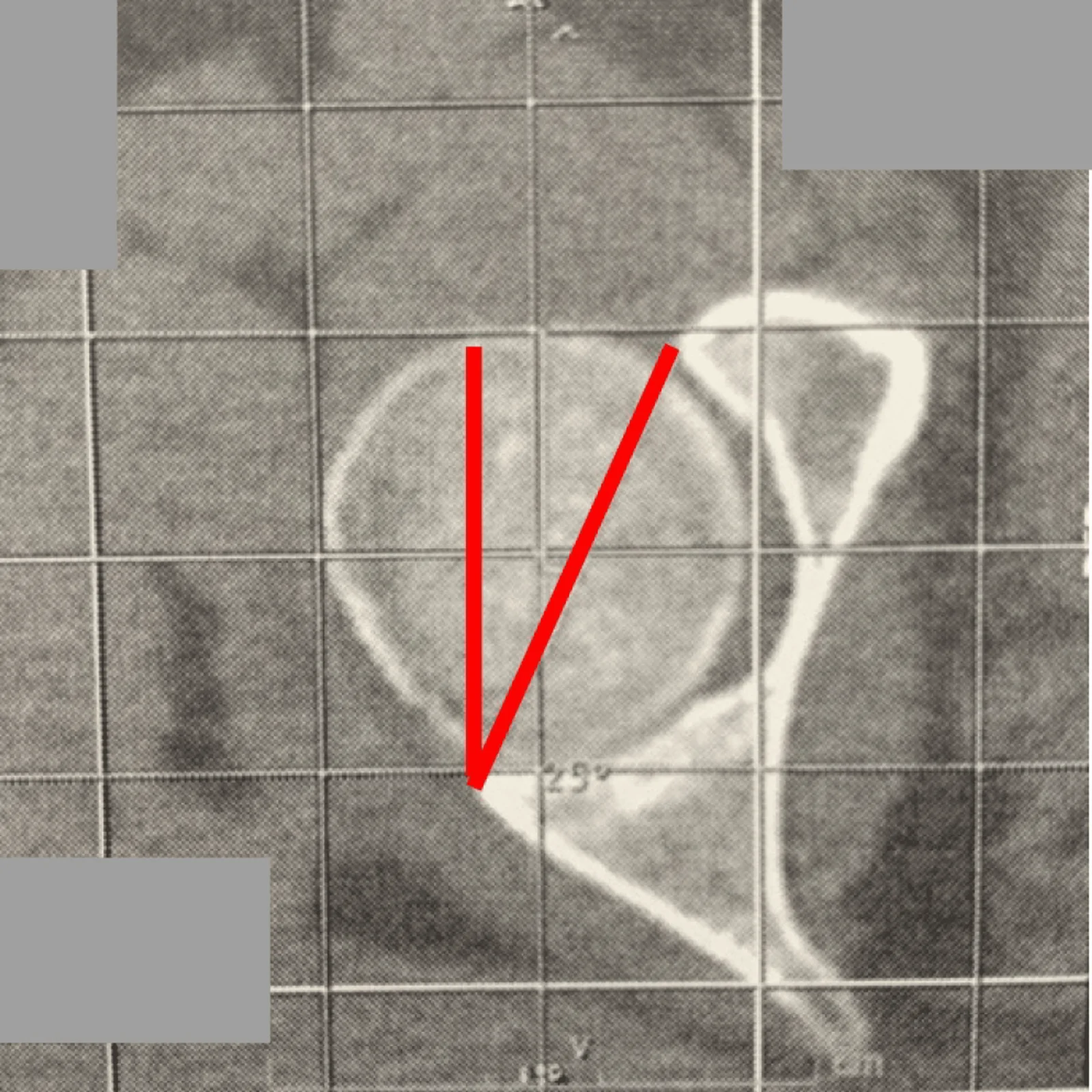
Measurement of acetabular anteversion Measurement of acetabular anteversion in the APP-standardized coordinate system. Acetabular anteversion was defined as the angle between the line connecting the anterior and posterior acetabular rims and the sagittal plane of the standardized anatomic pelvic plane-based coordinate system in the axial plane passing through the femoral head center. The reference sagittal plane is an APP-based anatomical reference and does not represent the functional pelvic plane APP: anatomic pelvic plane

All measurements were performed after transformation to the APP-based coordinate system to minimize variation attributable to pelvic orientation during CT acquisition.

Regional femoral head coverage

Regional acetabular coverage of the femoral head was evaluated using the AASA, PASA, and HASA. On the axial plane passing through the center of the femoral head, the AASA was defined as the angle between the line connecting the femoral head center to the anterior acetabular rim and the coronal plane of the standardized pelvic coordinate system. Representative measurements are shown for the right hip (Figure 8A) and left hip (Figure 8B). The PASA was defined as the corresponding angle between the line connecting the femoral head center to the posterior acetabular rim and the coronal plane. Representative measurements are likewise shown for the right hip (Figure 9A) and left hip (Figure 9B).

**Figure 8:**
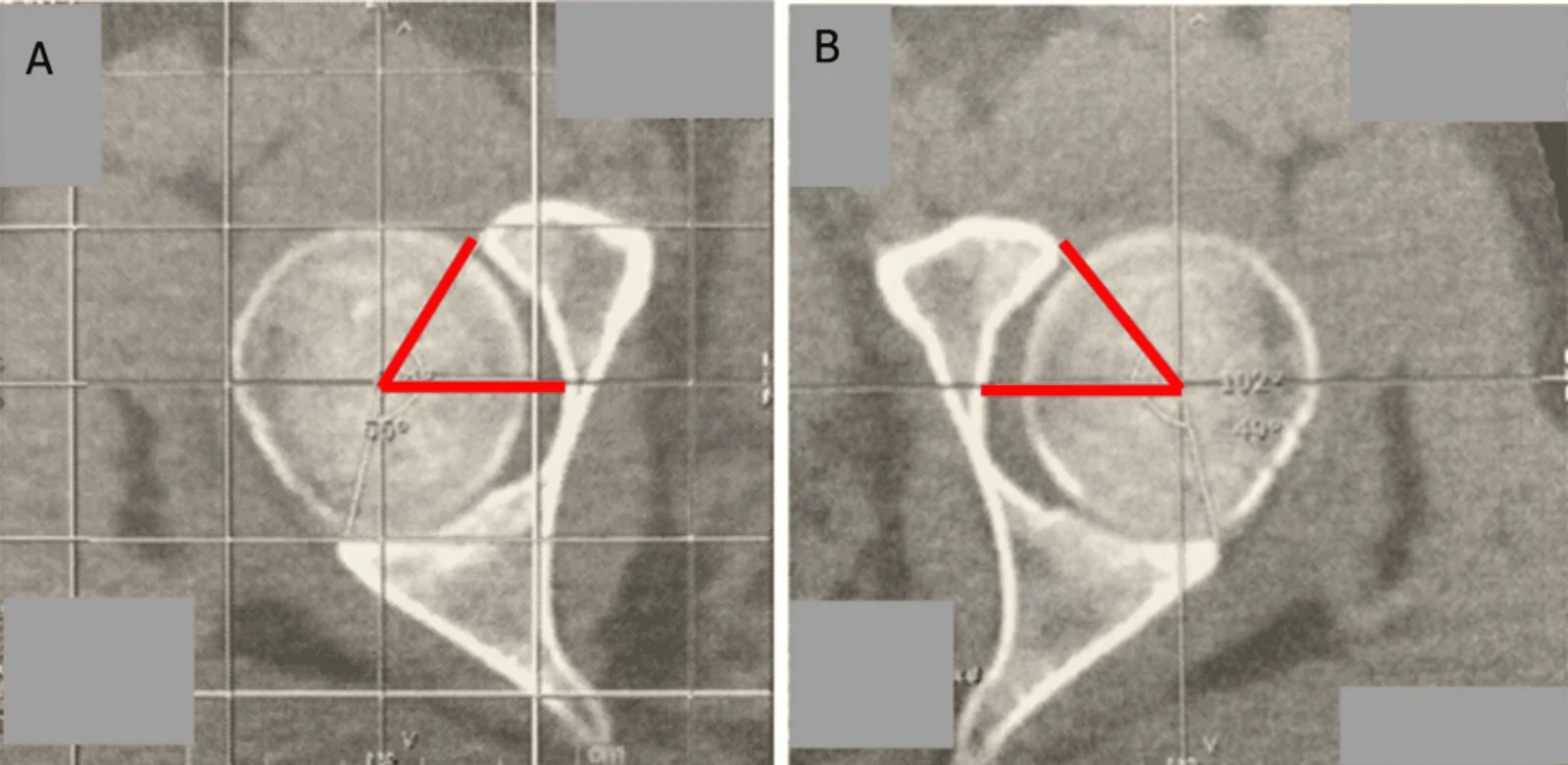
Measurement of the anterior acetabular sector angle. (A) Right hip. (B) Left hip. The anterior acetabular sector angle was defined as the angle between the line connecting the femoral head center to the anterior acetabular rim and the coronal plane on the axial plane passing through the femoral head center.

**Figure 9:**
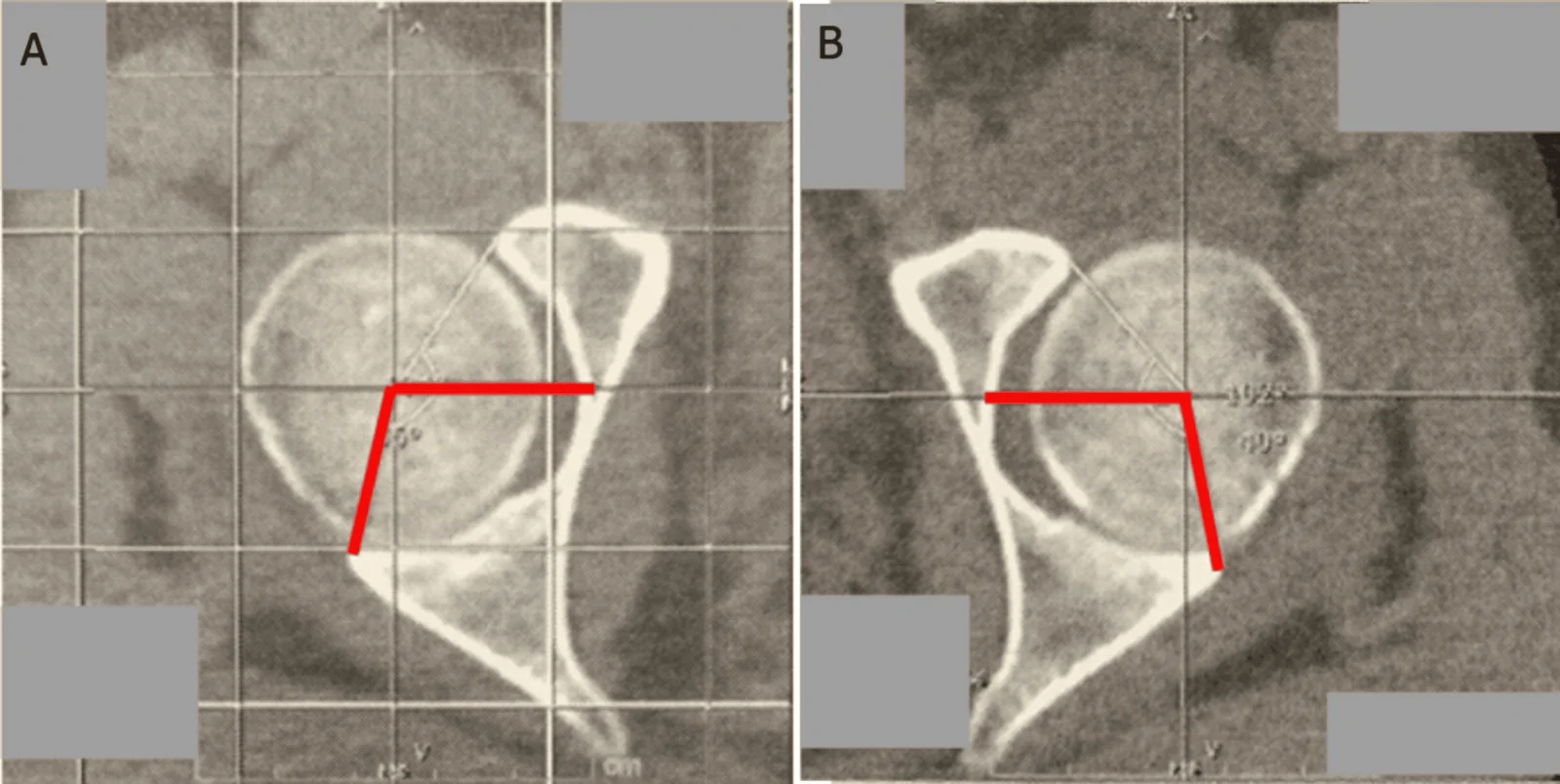
Measurement of the posterior acetabular sector angle (A) Right hip. (B) Left hip. The posterior acetabular sector angle was defined as the angle between the line connecting the femoral head center to the posterior acetabular rim and the coronal plane on the axial plane passing through the femoral head center

The HASA was calculated as the sum of the AASA and PASA and was used as an overall measure of horizontal acetabular coverage.

Conventional morphometric parameters

In addition to the pelvis-based three-dimensional measurements, conventional morphometric parameters were assessed on the standardized CT images, including femoral head diameter, CE angle, Sharp angle, and acetabular head index (AHI).

To measure the CE angle, we first identified the femoral head center on the sagittal slice showing the maximum femoral head diameter. The femoral head center was then aligned on the axial and coronal planes. On the coronal image, the CE angle was defined as the angle between a line passing through the femoral head center perpendicular to the line connecting the inferior margins of the bilateral teardrops and a line connecting the femoral head center to the lateral acetabular rim.

The Sharp angle was measured on the coronal image as the angle between the line connecting the inferior margins of the bilateral teardrops and the line connecting the inferior margin of the teardrop to the lateral acetabular rim.

The AHI was calculated as the distance from the medial margin of the femoral head to the lateral acetabular rim divided by the transverse diameter of the femoral head and multiplied by 100. Femoral head diameter was measured in millimeters by identifying the maximum diameter on each of the axial, coronal, and sagittal CT planes and using the largest of these measurements as the femoral head diameter. These conventional parameters were retained as complementary morphometric variables and were not used as primary eligibility criteria for the reference cohort.

Coverage area measurements

Coverage areas were quantified using software-assisted analysis of standardized three-dimensional CT reconstructions. The pelvic and femoral head components were segmented separately using Zio Station 2+ (Ziosoft, Tokyo, Japan) and subsequently imported into AW Volume Share 5 (GE Healthcare), where the reconstructed pelvis and femoral heads were superimposed and displayed with different rendering intensities.

After standardization to the APP-based coordinate system, the centers of both femoral heads were identified using the manual cross-grid method described above. The pelvis was then oriented so that the bilateral femoral head centers were horizontally aligned. A horizontal plane passing through the centers of both femoral heads was established. The portion of each femoral head below this plane was excluded, and the portion above the plane was retained for coverage-area analysis (Figures 10A, 10B).

**Figure 10:**
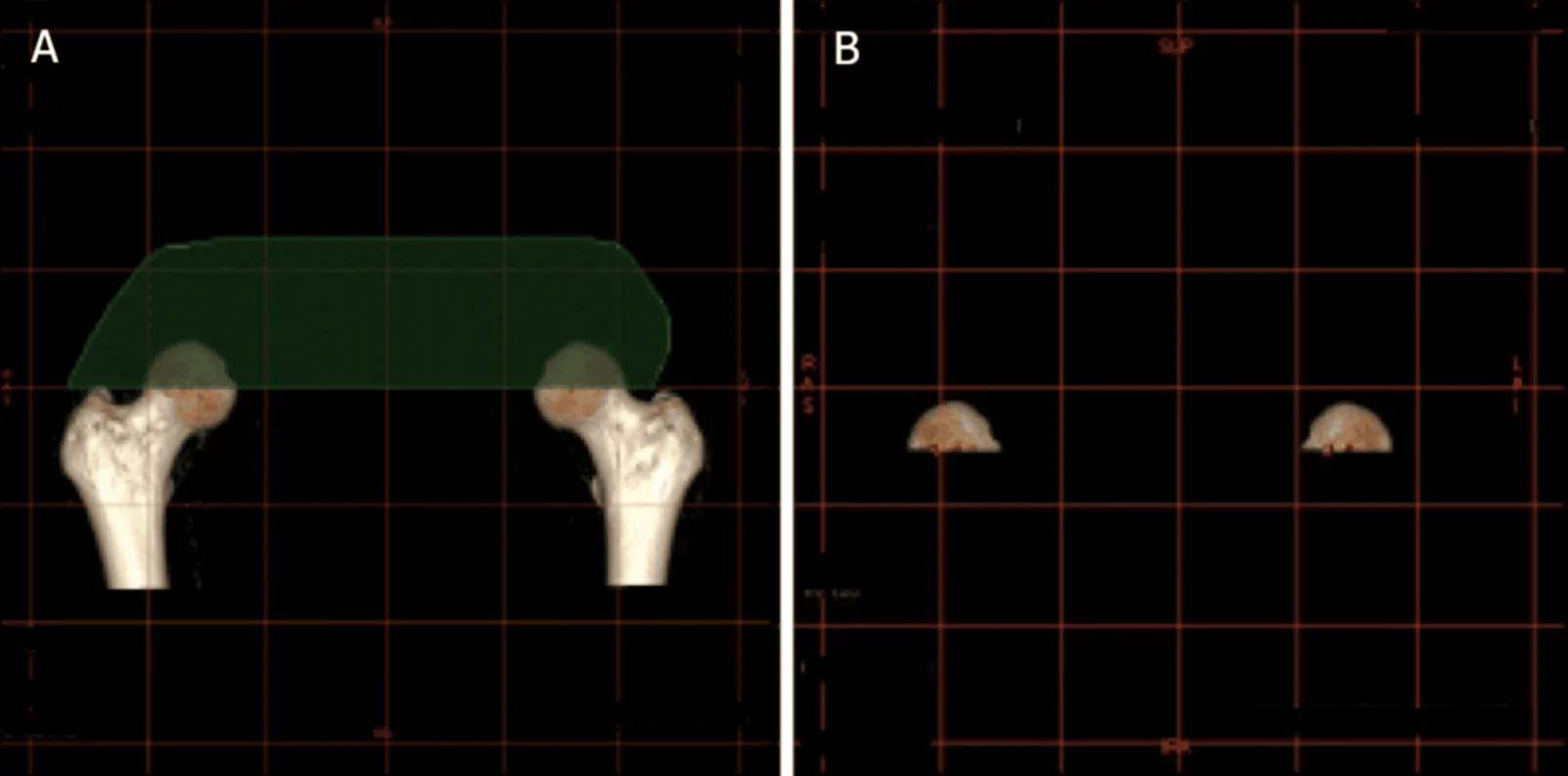
Measurement of anterior femoral head coverage area (A) After standardization to the anatomic pelvic plane, a horizontal plane passing through the centers of both femoral heads was established. The portion of each femoral head below this plane was excluded, and the portion above the plane was retained for coverage-area analysis. (B) The retained superior portion of each femoral head was isolated for projection-based measurement. On the standardized frontal projection, the region of the projected femoral head overlapped by the acetabulum was delineated and measured in square millimeters. The measurement represents a two-dimensional projected area derived from the standardized three-dimensional CT reconstruction rather than a true three-dimensional surface area

Coverage areas were quantified as two-dimensional projected areas derived from the standardized three-dimensional CT reconstructions and did not represent true three-dimensional mesh surface areas. For overall coverage, the retained superior portion of the femoral head was projected in the standardized APP-based orientation. The entire projected region of the retained femoral head overlapped by the acetabulum was delineated using the image-analysis software, and its area was recorded in square millimeters.

Anterior and posterior coverage areas were measured separately. For anterior coverage, the standardized reconstruction was viewed from the anterior direction, and the acetabular-covered region of the retained femoral head was isolated on the corresponding projection and measured in square millimeters (Figure 10). For posterior coverage, the reconstruction was viewed from the posterior direction, and the corresponding acetabular-covered region of the retained femoral head was similarly isolated and measured (Figure 11).

**Figure 11:**
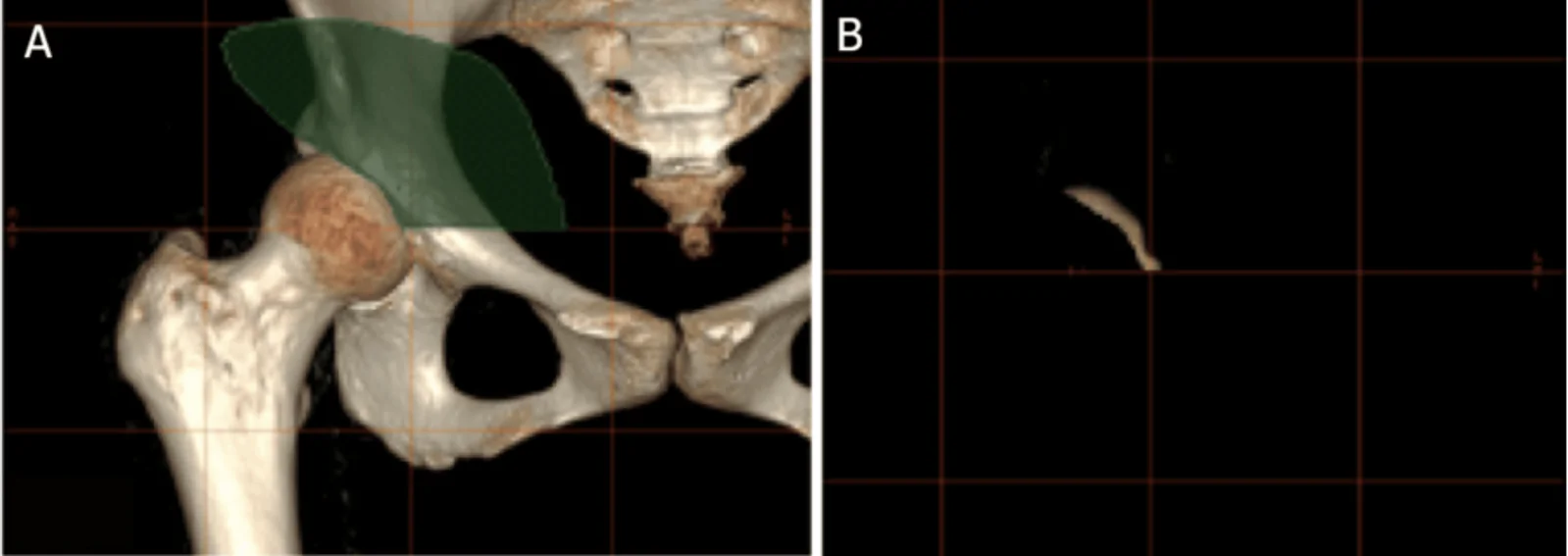
Measurement of posterior femoral head coverage area (A) Posterior view of the standardized three-dimensional reconstruction after exclusion of the portion of the femoral head below the horizontal plane passing through the bilateral femoral head centers. The superior portion of the femoral head was retained for coverage-area analysis. (B) The acetabular-covered region of the retained femoral head was isolated on the standardized posterior projection and measured in square millimeters. The measurement represents a two-dimensional projected area derived from the standardized three-dimensional CT reconstruction rather than a true three-dimensional surface area.

The anterior, posterior, and overall coverage measurements were not defined as mutually exclusive three-dimensional surface sectors, and no separate superior coverage area was measured. The overall coverage area was measured independently by delineating the entire acetabular-covered projected region of the retained femoral head and was not calculated as the arithmetic sum of the anterior and posterior coverage areas. Accordingly, overall, anterior, and posterior coverage areas were recorded as separate projection-based measurements in square millimeters.

Measurement reliability

Intraobserver reliability was assessed in 25 participants. For the originally evaluated morphometric parameters, the same observer repeated the measurements after a two-week interval. In response to the reliability assessment of the coverage-area measurements, the same observer also repeated the overall, anterior, and posterior projection-based coverage-area measurements in the same 25 participants. Reliability was evaluated separately for the right and left hips using intraclass correlation coefficients (ICCs) based on a two-way mixed-effects model for absolute agreement and single measurements. ICCs with 95% confidence intervals were reported.

Statistical analysis

Continuous variables were summarized as mean ± standard deviation, minimum and maximum values, and 95% confidence intervals of the mean. For anatomical reference measures, 95% reference intervals were estimated using the empirical 2.5th and 97.5th percentiles of the observed distributions.

Unadjusted relationships among the principal pelvis-based morphometric parameters were described using Pearson correlation coefficients and were considered descriptive because bilateral hips were included. Effects of laterality and age were evaluated using linear mixed-effects models with participant ID as a random intercept and side and age as fixed effects. Age was treated as a continuous variable, and the laterality effect was defined as the estimated left-minus-right difference. Side-by-age interactions were additionally examined for LI, AI, AV, AASA, PASA, and HASA after mean-centering age. p values for the six interaction tests were adjusted using the Benjamini-Hochberg false discovery rate procedure. As an additional exploratory sensitivity analysis, participants were stratified by age decade into 19-29, 30-39, 40-49, and 50-59 years. Age-decade effects on the principal pelvis-based morphometric parameters and coverage-area measurements were evaluated using linear mixed-effects models with participant ID as a random intercept and age-decade group and side as fixed effects. Overall p values for age-decade group were obtained using a joint test of the categorical age-decade terms.

Associations among the principal morphometric parameters were evaluated using generalized estimating equations with participant ID as the clustering variable and adjustment for age and side. Continuous predictor and outcome variables were standardized to z-scores before model fitting, and standardized regression coefficients (β) with 95% confidence intervals and p values were reported. The GEE association analyses were considered exploratory, and no multiplicity adjustment was applied to these association tests. False discovery rate adjustment was applied only to the six side-by-age interaction tests described above.

The CE angle was not used as a primary eligibility criterion. As a sensitivity analysis, the principal associations were re-evaluated in hips with a CT-based CE angle of 25°-40°, representing a conventionally used range of lateral acetabular coverage rather than a universal biological normal range [10]. CT-based CE angle measurements have been shown to correlate with conventional radiographic measurements [11]. This subgroup was analyzed using generalized estimating equations adjusted for age and side. A second sensitivity analysis was performed using the right hip only, with associations evaluated using age-adjusted linear regression.

All statistical tests were two-sided, with p < 0.05 considered statistically significant. For the side-by-age interaction analyses, false discovery rate-adjusted q values were additionally reported. Statistical analyses were performed using IBM Statistical Package for the Social Sciences Statistics, version 25.0 (IBM Corp., Armonk, NY) and Stata, version 14.1 (StataCorp LP, College Station, TX).

## Results

Study cohort and morphometric distribution

The study cohort comprised 172 Japanese women with bilateral measurements available for all participants, yielding 344 hips for analysis. The mean age at CT acquisition was 45.8 ± 10.7 years (range, 19-59 years).

The mean lateralization index was 68.4 ± 5.7, and the mean anterior index was 62.5 ± 3.3. The mean AV, AASA, PASA, and HASA were 22.0° ± 6.4°, 57.3° ± 8.3°, 99.5° ± 9.0°, and 156.8° ± 10.0°, respectively. The mean femoral head diameter was 41.9 ± 2.2 mm. The mean CE angle was 34.3° ± 6.0°, the mean Sharp angle was 41.4° ± 3.8°, and the mean AHI was 90.6% ± 6.6%.

The mean overall, anterior, and posterior coverage areas were 697.4 ± 79.0, 232.8 ± 76.3, and 508.5 ± 75.3 mm², respectively. The distributions and corresponding 95% reference intervals of the evaluated morphometric parameters are summarized in Table 1.

**Table 1:** Descriptive statistics of the evaluated morphometric parameters Values are based on 344 hips from 172 participants. The 95% reference intervals represent the empirical 2.5th and 97.5th percentiles of the observed distributions SD: standard deviation; CI: confidence interval

Parameter	Mean ± SD	95% CI of mean	Minimum-maximum	95% reference interval
Lateralization index	68.4 ± 5.7	67.8-69.0	56.6-85.5	59.9-81.7
Anterior index	62.5 ± 3.3	62.2-62.9	50.0-70.2	55.1-68.6
Acetabular anteversion (°)	22.0 ± 6.4	21.4-22.7	6-38	11-35
Anterior acetabular sector angle (°)	57.3 ± 8.3	56.5-58.2	38-106	43.0-71.4
Posterior acetabular sector angle (°)	99.5 ± 9.0	98.5-100.4	66-126	83.0-117.9
Horizontal acetabular sector angle (°)	156.8 ± 10.0	155.8-157.9	126-191	140.6-180.4
Femoral head diameter (mm)	41.9 ± 2.2	41.6-42.1	35.6-49.2	37.7-46.1
Center-edge angle (°)	34.3 ± 6.0	33.7-35.0	15-60	24-48
Sharp angle (°)	41.4 ± 3.8	41.0-41.8	30-51	33.0-48.4
Acetabular head index (%)	90.6 ± 6.6	89.9-91.3	70.5-99.5	77.3-99.2
Overall coverage area (mm²)	697.4 ± 79.0	689.0-705.8	508.2-1019.0	555.2-882.2
Anterior coverage area (mm²)	232.8 ± 76.3	224.7-240.9	29.2-510.2	99.1-394.0
Posterior coverage area (mm²)	508.5 ± 75.3	500.5-516.5	318.6-699.4	365.7-671.1

Correlations among three-dimensional morphometric parameters

In the unadjusted analysis, AI was positively correlated with AV (r = 0.66) and PASA (r = 0.45) and negatively correlated with AASA (r = -0.50), whereas essentially no correlation was observed between AI and HASA (r = -0.01) (Table 2). The LI showed smaller correlations with the evaluated acetabular parameters. AV was negatively correlated with AASA (r = -0.67) and positively correlated with PASA (r = 0.76).

**Table 2:** Unadjusted correlations among the principal pelvis-based three-dimensional morphometric parameters Values are unadjusted Pearson correlation coefficients calculated using all 344 hips. These coefficients are presented descriptively; inferential associations accounting for within-participant clustering and adjustment for age and side are presented in Table 3 AI: anterior index; LI: lateralization index; AV: acetabular anteversion; AASA: anterior acetabular sector angle; PASA: posterior acetabular sector angle; HASA: horizontal acetabular sector angle

Parameter	AI	LI	AV	AASA	PASA	HASA
AI	-	0.27	0.66	-0.50	0.45	-0.01
LI	-	-	0.32	-0.32	0.17	-0.11
AV	-	-	-	-0.67	0.76	0.13
AASA	-	-	-	-	-0.34	0.53
PASA	-	-	-	-	-	0.62
HASA	-	-	-	-	-	-

**Table 3:** Cluster-adjusted associations among pelvis-based three-dimensional morphometric parameters Standardized coefficients were estimated using generalized estimating equations with participant ID as the clustering variable and adjustment for age and side β: standardized regression coefficient; CI: confidence interval; AI: anterior index; LI: lateralization index; AV: acetabular anteversion; AASA: anterior acetabular sector angle; PASA: posterior acetabular sector angle; HASA: horizontal acetabular sector angle

Predictor	Outcome	Standardized β	95% CI	p value
AI	AV	0.62	0.53 to 0.70	<0.001
	AASA	-0.40	-0.49 to -0.32	<0.001
	PASA	0.32	0.23 to 0.42	<0.001
	HASA	-0.04	-0.13 to 0.06	0.43
LI	AV	0.28	0.16 to 0.41	<0.001
	AASA	-0.30	-0.41 to -0.19	<0.001
	PASA	0.11	-0.01 to 0.24	0.08
	HASA	-0.16	-0.28 to -0.04	0.009
AV	AASA	-0.67	-0.75 to -0.59	<0.001
	PASA	0.69	0.61 to 0.76	<0.001
	HASA	0.05	-0.03 to 0.13	0.24

Cluster-adjusted associations among three-dimensional morphometric parameters

After accounting for clustering of bilateral hips within participants and adjusting for age and side, the principal associations observed in the unadjusted analysis remained evident (Table 3). The AI was positively associated with AV (standardized β = 0.62; 95% CI, 0.53-0.70; p < 0.001), negatively associated with AASA (β = -0.40; 95% CI, -0.49 to -0.32; p < 0.001), and positively associated with PASA (β = 0.32; 95% CI, 0.23-0.42; p < 0.001). No significant association was observed between AI and HASA (β = -0.04; 95% CI, -0.13 to 0.06; p = 0.43).

The LI showed weaker associations with acetabular orientation and regional coverage. LI was positively associated with AV (β = 0.28; 95% CI, 0.16-0.41; p < 0.001) and negatively associated with AASA (β = -0.30; 95% CI, -0.41 to -0.19; p < 0.001), whereas its association with PASA did not reach statistical significance (β = 0.11; 95% CI, -0.01 to 0.24; p = 0.08). LI was also negatively associated with HASA (β = -0.16; 95% CI, -0.28 to -0.04; p = 0.009).

AV was strongly negatively associated with AASA (β = -0.67; 95% CI, -0.75 to -0.59; p < 0.001) and strongly positively associated with PASA (β = 0.69; 95% CI, 0.61-0.76; p < 0.001), whereas no significant association was observed between AV and HASA (β = 0.05; 95% CI, -0.03 to 0.13; p = 0.24).

Effects of laterality and age

No statistically significant side-to-side differences were observed in the principal pelvis-based three-dimensional morphometric parameters after accounting for within-participant correlation (Table 4). No significant associations with age were identified for LI, AI, AV, AASA, PASA, or HASA.

**Table 4:** Linear mixed-effects model analysis of laterality and age for morphometric parameters ^*^Laterality was encoded as the left value minus the right value; therefore, positive values indicate higher values on the left side, and negative values indicate higher values on the right side. Age estimates represent the change in each parameter per one-year increase in age. Linear mixed-effects models included participant ID as a random intercept, with side and age as fixed effects CI: confidence interval; LI: lateralization index; AI: anterior index; AV: acetabular anteversion; AASA: anterior acetabular sector angle; PASA: posterior acetabular sector angle; HASA: horizontal acetabular sector angle; AHI: acetabular head index

Parameter	Left-right effect^*^	95% CI	p value	Age effect per year	95% CI	p value
LI	+0.13	-0.25 to 0.50	0.50	+0.04	-0.04 to 0.12	0.30
AI	+0.08	-0.27 to 0.43	0.65	+0.01	-0.04 to 0.05	0.76
AV (°)	-0.08	-0.90 to 0.74	0.85	+0.04	-0.04 to 0.12	0.31
AASA (°)	-0.09	-1.01 to 0.83	0.85	+0.01	-0.09 to 0.12	0.80
PASA (°)	-0.58	-1.51 to 0.35	0.22	+0.06	-0.06 to 0.17	0.35
HASA (°)	-0.67	-1.50 to 0.16	0.12	+0.07	-0.06 to 0.21	0.30
Femoral head diameter (mm)	-0.36	-0.53 to -0.18	<0.001	+0.045	0.016 to 0.073	0.002
AHI (%)	-0.45	-0.88 to -0.01	0.044	+0.091	0.001 to 0.181	0.047
Overall coverage area (mm²)	-11.78	-17.62 to -5.95	<0.001	+1.63	0.59 to 2.68	0.002
Anterior coverage area (mm²)	-8.89	-15.64 to -2.14	0.010	+0.46	-0.56 to 1.48	0.38
Posterior coverage area (mm²)	+1.40	-5.10 to 7.89	0.67	+1.43	0.43 to 2.42	0.005

In contrast, several complementary morphometric measures showed modest side- or age-related differences. Femoral head diameter, AHI, overall coverage area, and anterior coverage area were greater on the right than on the left. Femoral head diameter, AHI, overall coverage area, and posterior coverage area showed positive associations with age.

Side-by-age interactions were additionally examined for the six principal pelvis-based three-dimensional morphometric parameters (Table 5). A nominally significant interaction was observed for PASA, with the left-right difference increasing by 0.097° per one-year increase in age (95% CI, 0.010-0.183; p = 0.028). However, this interaction did not remain statistically significant after Benjamini-Hochberg false discovery rate adjustment (q = 0.170). No significant side-by-age interactions were identified for LI, AI, AV, AASA, or HASA.

**Table 5:** Linear mixed-effects model analysis of side-by-age interactions for pelvis-based three-dimensional morphometric parameters ^*^Interaction estimates represent the change in the left-right difference per one-year increase in age. Linear mixed-effects models included participant ID as a random intercept, with side, mean-centered age, and the side-by-age interaction as fixed effects. False discovery rate adjustment was applied to the six interaction tests using the Benjamini-Hochberg procedure CI: confidence interval; q: false discovery rate-adjusted p value; LI: lateralization index; AI: anterior index; AV: acetabular anteversion; AASA: anterior acetabular sector angle; PASA: posterior acetabular sector angle; HASA: horizontal acetabular sector angle

Parameter	Side × age interaction estimate^*^	95% CI	p value	FDR-adjusted q value
LI	+0.032 per year	-0.003 to 0.067	0.074	0.222
AI	-0.021 per year	-0.054 to 0.011	0.202	0.324
AV (°)	+0.035 per year	-0.042 to 0.112	0.374	0.374
AASA (°)	-0.049 per year	-0.135 to 0.038	0.270	0.324
PASA (°)	+0.097 per year	0.010 to 0.183	0.028	0.170
HASA (°)	+0.048 per year	-0.030 to 0.126	0.226	0.324

Sensitivity analyses

Sensitivity analyses were performed to assess whether the principal morphometric associations depended on the definition of conventional lateral acetabular coverage or on the inclusion of bilateral hips. Of the 344 hips included in the primary cohort, 10 hips (2.9%) had a CT-based CE angle <25°, 284 hips (82.6%) had a CE angle of 25°-40°, and 50 hips (14.5%) had a CE angle >40°. The 284 hips within the 25°-40° range were included in the sensitivity analysis restricted to conventionally normal lateral acetabular coverage.

The principal associations were preserved within this subgroup (Table 6). The association between AI and AV remained positive (standardized β = 0.65; 95% CI, 0.55-0.74; p < 0.001), whereas AI remained negatively associated with AASA (β = -0.51; 95% CI, -0.60 to -0.42; p < 0.001) and positively associated with PASA (β = 0.50; 95% CI, 0.40-0.60; p < 0.001). The associations of AV with AASA and PASA were also preserved.

**Table 6:** Generalized estimating equation analysis restricted to hips with a center-edge angle of 25°-40° The analysis included 284 hips with a center-edge angle of 25°-40°. Standardized coefficients were estimated using generalized estimating equations with participant ID as the clustering variable and adjustment for age and side β: standardized regression coefficient; CI: confidence interval; CE: center-edge; LI: lateralization index; AI: anterior index; AV: acetabular anteversion; AASA: anterior acetabular sector angle; PASA: posterior acetabular sector angle

Predictor	Outcome	Primary cohort standardized β	CE 25°-40° subgroup standardized β	95% CI	p value
AI	AV	0.62	0.65	0.55 to 0.74	<0.001
	AASA	-0.40	-0.51	-0.60 to -0.42	<0.001
	PASA	0.32	0.50	0.40 to 0.60	<0.001
LI	AV	0.28	0.31	0.18 to 0.44	<0.001
	AASA	-0.30	-0.34	-0.47 to -0.21	<0.001
AV	AASA	-0.67	-0.77	-0.85 to -0.68	<0.001
	PASA	0.69	0.76	0.68 to 0.84	<0.001

A second sensitivity analysis using only the right hip from each participant (n = 172) yielded similar results (Table 7). AI remained positively associated with AV (standardized β = 0.66; 95% CI, 0.55-0.78; p < 0.001), negatively associated with AASA (β = -0.47; 95% CI, -0.61 to -0.34; p < 0.001), and positively associated with PASA (β = 0.43; 95% CI, 0.29-0.57; p < 0.001). Thus, the principal morphometric associations were not materially altered by restriction according to conventional lateral coverage or by analysis of one hip per participant.

**Table 7:** Age-adjusted linear regression analysis restricted to right hips The analysis included one right hip from each participant (n = 172). Standardized coefficients were estimated using age-adjusted linear regression models β: standardized regression coefficient; CI: confidence interval; LI: lateralization index; AI: anterior index; AV: acetabular anteversion; AASA: anterior acetabular sector angle; PASA: posterior acetabular sector angle

Predictor	Outcome	Bilateral primary analysis standardized β	Right-hip-only standardized β	95% CI	p value
AI	AV	0.62	0.66	0.55 to 0.78	<0.001
	AASA	-0.40	-0.47	-0.61 to -0.34	<0.001
	PASA	0.32	0.43	0.29 to 0.57	<0.001
LI	AV	0.28	0.27	0.13 to 0.42	<0.001
	AASA	-0.30	-0.32	-0.47 to -0.18	<0.001
AV	AASA	-0.67	-0.66	-0.78 to -0.55	<0.001
	PASA	0.69	0.78	0.68 to 0.88	<0.001

Reference distributions were additionally calculated for the 284 hips with a CT-based CE angle of 25°-40° using the same empirical percentile method applied to the primary cohort (Table 8). The subgroup reference intervals were generally similar to those observed in the primary cohort.

**Table 8:** Reference distributions of morphometric parameters in hips with a CT-based center-edge angle of 25°-40° Values are based on 284 hips with a CT-based center-edge angle of 25°-40°. The 95% reference intervals represent the empirical 2.5th and 97.5th percentiles of the observed distributions CT: computed tomography; SD: standard deviation

Parameter	Mean ± SD	Minimum-maximum	95% reference interval
Lateralization index	68.5 ± 5.9	56.6-85.5	59.8-82.4
Anterior index	62.5 ± 3.4	50.0-70.2	55.3-68.7
Acetabular anteversion (°)	21.8 ± 6.4	6-38	11.0-34.0
Anterior acetabular sector angle (°)	56.8 ± 7.4	38-77	42.1-70.9
Posterior acetabular sector angle (°)	98.8 ± 8.1	74-120	83.1-115.0
Horizontal acetabular sector angle (°)	155.6 ± 9.1	126-184	140.0-175.9
Femoral head diameter (mm)	41.9 ± 2.1	35.6-49.2	37.9-45.8
Center-edge angle (°)	32.9 ± 3.8	25-40	25.0-40.0
Sharp angle (°)	41.9 ± 3.3	32-51	35.0-48.9
Acetabular head index (%)	90.5 ± 6.7	73.9-99.5	77.3-99.2
Overall coverage area (mm²)	699.7 ± 80.4	508.2-1,019.0	553.4-901.6
Anterior coverage area (mm²)	230.0 ± 75.2	29.2-510.2	99.5-382.8
Posterior coverage area (mm²)	503.6 ± 73.8	318.6-696.3	363.4-664.1

Intraobserver reliability

Intraobserver reliability was high across the evaluated morphometric measurements, with ICCs ranging from 0.810 to 0.997 across the right and left hips (Table 9). For the projection-based coverage-area measurements, ICCs ranged from 0.810 to 0.953. Overall coverage area showed ICCs of 0.892 (95% CI, 0.772-0.951) for the right hip and 0.810 (95% CI, 0.616-0.912) for the left hip. Anterior coverage area showed ICCs of 0.937 (95% CI, 0.847-0.973) and 0.953 (95% CI, 0.891-0.980), respectively, whereas posterior coverage area showed ICCs of 0.929 (95% CI, 0.847-0.968) and 0.858 (95% CI, 0.707-0.934), respectively. Detailed ICC estimates and 95% confidence intervals are provided in Table 9.

**Table 9:** Intraobserver reliability of morphometric measurements Intraobserver reliability was assessed in 25 participants using repeated measurements by the same observer. Intraclass correlation coefficients were calculated separately for the right and left hips using a two-way mixed-effects model for absolute agreement based on single measurements, with 95% confidence intervals. The overall, anterior, and posterior coverage-area measurements represent projection-based areas derived from standardized three-dimensional CT reconstructions ICC: intraclass correlation coefficient; CI: confidence interval; LI: lateralization index; AI: anterior index; AV: acetabular anteversion; AASA: anterior acetabular sector angle; PASA: posterior acetabular sector angle; HASA: horizontal acetabular sector angle; CE: center-edge; AHI: acetabular head index

Parameter	Right hip ICC	95% CI	Left hip ICC	95% CI
LI	0.987	0.971-0.994	0.993	0.984-0.997
AI	0.972	0.937-0.987	0.985	0.963-0.993
AV	0.993	0.983-0.997	0.915	0.817-0.962
AASA	0.995	0.989-0.998	0.997	0.993-0.999
PASA	0.995	0.988-0.998	0.996	0.990-0.998
HASA	0.987	0.971-0.994	0.988	0.970-0.995
Femoral head diameter	0.966	0.916-0.986	0.978	0.938-0.991
CE angle	0.975	0.945-0.989	0.979	0.953-0.991
Sharp angle	0.910	0.805-0.959	0.957	0.906-0.981
AHI	0.945	0.879-0.976	0.946	0.881-0.976
Overall coverage area	0.892	0.772-0.951	0.810	0.616-0.912
Anterior coverage area	0.937	0.847-0.973	0.953	0.891-0.980
Posterior coverage area	0.929	0.847-0.968	0.858	0.707-0.934

Age-decade sensitivity analysis

An additional exploratory sensitivity analysis was performed after stratifying participants by age decade into 19-29 years (22 participants, 44 hips), 30-39 years (23 participants, 46 hips), 40-49 years (48 participants, 96 hips), and 50-59 years (79 participants, 158 hips) (Table 10). No significant age-decade effect was observed for the six principal pelvis-based positional and angular parameters, including LI, AI, AV, AASA, PASA, and HASA (all p ≥ 0.193). In contrast, overall coverage area differed significantly across age decades (p = 0.011), as did posterior coverage area (p = 0.030), whereas anterior coverage area did not (p = 0.327). These findings were consistent with the primary continuous-age analysis, which showed relative stability of the principal pelvis-based positional and angular parameters within the observed age range, while selected coverage-area measurements showed age-related variation.

**Table 10:** Exploratory age-decade sensitivity analysis of principal pelvis-based three-dimensional morphometric parameters and coverage areas Values are presented as mean ± standard deviation. Participants were stratified into four age-decade groups: 19-29 years (22 participants, 44 hips), 30-39 years (23 participants, 46 hips), 40-49 years (48 participants, 96 hips), and 50-59 years (79 participants, 158 hips). Overall age-decade effects were evaluated using linear mixed-effects models with participant ID as a random intercept and age-decade group and side as fixed effects. p values represent the overall test of the categorical age-decade effect

Parameter	19-29 years (n = 44 hips)	30-39 years (n = 46 hips)	40-49 years (n = 96 hips)	50-59 years (n = 158 hips)	p value
Lateralization index	68.1 ± 5.6	67.9 ± 4.4	67.7 ± 5.4	69.1 ± 6.2	0.564
Anterior index	63.1 ± 3.2	61.4 ± 4.0	62.3 ± 3.5	62.8 ± 3.0	0.193
Acetabular anteversion (°)	22.1 ± 5.8	20.6 ± 6.4	21.8 ± 6.2	22.6 ± 6.6	0.504
Anterior acetabular sector angle (°)	56.2 ± 6.2	58.3 ± 7.6	57.1 ± 7.5	57.5 ± 9.5	0.828
Posterior acetabular sector angle (°)	98.8 ± 6.9	98.5 ± 9.4	99.2 ± 7.4	100.2 ± 10.2	0.802
Horizontal acetabular sector angle (°)	155.0 ± 6.8	156.8 ± 11.4	156.3 ± 8.4	157.7 ± 11.1	0.671
Overall coverage area (mm²)	654.3 ± 77.4	695.3 ± 66.3	692.0 ± 65.4	713.3 ± 85.8	0.011
Anterior coverage area (mm²)	210.9 ± 71.5	249.4 ± 83.0	229.4 ± 78.0	236.1 ± 73.7	0.327
Posterior coverage area (mm²)	474.8 ± 84.0	502.7 ± 70.0	502.2 ± 63.1	523.3 ± 78.0	0.030

## Discussion

The present study characterized pelvis-based three-dimensional hip morphology in 172 Japanese women (344 hips) without clinically recognized hip disease and identified two principal findings. First, the anterior position of the femoral head within the pelvis, represented by the AI, was consistently associated with acetabular orientation and regional coverage: a greater AI was associated with greater AV, reduced anterior sector coverage, and greater posterior sector coverage. In contrast, the LI showed substantially weaker associations with these acetabular parameters. Second, the principal pelvis-based positional and angular parameters showed no material evidence of variation by side or age within the observed age range, and the major morphometric associations were preserved both in hips with a CT-based CE angle of 25°-40° and in the right-hip-only analysis. Together, these findings indicate that femoral head position within the pelvis is related to the three-dimensional orientation and distribution of acetabular coverage and support evaluation of the hip as an integrated pelvis-femoral head-acetabulum spatial relationship rather than solely by conventional coronal coverage measures.

The most notable finding of the present study was a consistent association between the pelvis-relative anterior position of the femoral head and acetabular orientation and regional coverage. A greater AI was associated with greater AV, smaller AASA, and larger PASA, and these relationships persisted after accounting for bilateral clustering, age, and side. In an earlier CT study of hips considered radiographically normal, Yoshida et al. quantified femoral head position using the AI and LI together with AV and sector angles and reported a positive relationship between AI and AV, providing an early indication that femoral head position within the pelvis and acetabular orientation are geometrically related [1]. Subsequent studies from the same group extended this concept to pathologic hips. Ishibashi et al. demonstrated that hips with secondary osteoarthritis associated with acetabular dysplasia were positioned more anterolaterally than reference hips and that progressive disease was accompanied by increasing lateralization and decreasing AV [5]. Henmi subsequently used three-dimensional CT to demonstrate that secondary coxarthrosis was characterized not only by lateral malposition but also by anterior malposition of the femoral head within the pelvis, together with regional differences in femoral head coverage [6]. These earlier observations are relevant to the present finding that anterior femoral head position was more closely related to acetabular orientation and anterior-posterior coverage than was lateral position. The present cross-sectional data cannot determine the direction of this relationship. Rather than indicating that anterior femoral head position causes AV, or vice versa, the observed association may reflect coordinated or reciprocal development of femoral head position and acetabular orientation within the pelvis. In this interpretation, both features represent components of an integrated pelvis-femoral head-acetabulum spatial configuration.

The relationship between acetabular orientation and regional coverage is also supported by independent studies. The acetabular sector-angle method was originally introduced to quantify anterior and posterior femoral head support on axial CT [12]. In dysplastic hips, increased posterior acetabular tilt has been associated with greater AV and reduced anterior and anterosuperior coverage [13]. Longitudinal imaging during adolescence has likewise shown that increasing AV is accompanied by decreasing anterior sector coverage [14]. Some of the observed correlations among the acetabular angular parameters should be interpreted in light of their shared geometric definitions. AV, AASA, and PASA are derived from related acetabular rim landmarks on the same axial plane, and HASA is defined as the sum of AASA and PASA; therefore, correlations among these parameters are partly mathematical by construction. In contrast, AI does not incorporate the acetabular rim landmarks and is defined independently from the PSIS-to-ASIS reference span. Thus, although shared use of the femoral head center and pelvic coordinate system may contribute some geometric dependence, the associations of AI with AV, AASA, and PASA are not algebraically predetermined. Taken together, the present findings support coordinated spatial relationships among femoral head position, acetabular orientation, and the anterior-posterior distribution of coverage, while the associations among the acetabular angular parameters themselves should not be interpreted as fully independent biological relationships. The present study extends this framework by demonstrating these relationships in a reference population of Japanese women without clinically recognized hip disease.

A second finding was the relative stability of the principal pelvis-based morphometric parameters with respect to laterality and age within the present cohort. No material side-to-side differences were identified for the AI, LI, AV, AASA, PASA, or HASA after accounting for within-participant correlation. This bilateral similarity is consistent with CT-based studies of asymptomatic or healthy adults reporting substantial symmetry of native hip and acetabular morphology [3,15]. The preservation of the principal associations in the right-hip-only analysis further indicates that these relationships were not driven by treating bilateral hips as independent observations.

In contrast, the absence of a clear age-related effect on the principal pelvis-based positional and angular parameters differs from several previous CT-based studies. In Japanese adults aged 30-88 years, Yamatani et al. reported age-related changes in CE angle, anterior coverage, Sharp angle, and AV [4]. Similarly, a large CT study of asymptomatic hips found increasing lateral coverage and acetabular version across age groups [3]. The present cohort was restricted to women aged 19-59 years without clinically recognized hip disease or overt degenerative or destructive abnormalities. This narrower age range and disease spectrum may have reduced the influence of advanced age-related degenerative or subchondral remodeling. However, menopausal status, bone mineral density, and direct markers of subchondral remodeling were not available in this retrospective cohort; therefore, the observed differences from previous studies cannot be attributed specifically to menopause or to a particular remodeling mechanism. Accordingly, the present findings should be interpreted as evidence of relative stability of the principal pelvis-based positional and angular parameters within the observed age range rather than as evidence that hip morphology is independent of aging more generally.

The present findings also have implications for how reference hip morphology is defined. Conventional radiographic parameters such as the CE and Sharp angles remain clinically useful, but a single coronal measurement cannot fully describe variation in femoral head position, acetabular orientation, or regional coverage. Reliance on the lateral CE angle alone may incompletely characterize acetabular morphology, particularly in hips near conventional thresholds of lateral coverage [16]. Moreover, reported reference distributions of the CE angle vary among study populations and imaging methods, supporting interpretation of such thresholds as conventional clinical references rather than universal anatomical boundaries [17,18]. CT-based studies of asymptomatic hips have similarly shown that acetabular coverage and version vary substantially even when conventional lateral coverage is within commonly accepted ranges [2]. Three-dimensional assessment is particularly relevant because pathologic hips may differ from controls not only in lateral coverage but also in the position of the femoral head relative to a pelvic coordinate system. Using an anterior pelvic plane-based coordinate system, Cheng et al. demonstrated three-dimensional displacement of the femoral head in developmental dysplasia of the hip compared with demographically matched controls [7]. Furthermore, recent three-dimensional analyses of hip dysplasia have demonstrated heterogeneous patterns of regional femoral head coverage that are not represented by lateral coverage alone [8]. Against this background, the distributions and interrelationships established in the present cohort may provide a population-specific anatomical reference framework for subsequent comparison with dysplastic, degenerative, or otherwise morphologically abnormal hips. Such a framework should be regarded as complementary to established radiographic measurements rather than as a diagnostic threshold, and its clinical relevance will require validation in cohorts with defined hip pathology and longitudinal outcomes.

The principal strengths of this study were the use of a standardized pelvis-based three-dimensional coordinate system, the inclusion of bilateral CT measurements from a relatively large cohort, and statistical analyses that accounted for within-participant correlation and tested the robustness of the principal findings in complementary sensitivity analyses. Several limitations should also be considered. First, this was a retrospective, single-center study restricted to Japanese women aged 19-59 years; therefore, the present reference distributions cannot be generalized directly to men, other ethnic populations, or older adults. Second, the CT examinations were obtained for nonorthopedic clinical indications rather than from prospectively recruited healthy volunteers. Although no participant had clinically recognized hip disease or overt degenerative or destructive abnormalities and no hips were excluded on the basis of a predefined CE-angle threshold, the cohort should therefore be regarded as a clinical reference population rather than an idealized healthy population. Third, all CT examinations were acquired in the supine position and subsequently standardized using the APP-based anatomical coordinate system. Although this approach provides a reproducible anatomical reference for comparing pelvis-relative hip morphology, it does not represent functional pelvic orientation during weight-bearing or changes in spinopelvic alignment with posture. Pelvic orientation is relevant to acetabular coverage; CT-based analysis has demonstrated a progressive reduction in femoral head coverage with increasing posterior pelvic tilt across dysplastic, borderline, and conventionally normal hips [10]. Anatomical pelvic morphology and position-dependent pelvic orientation should also be distinguished when considering sagittal alignment of the spine, pelvis, and lower extremities [19]. Methods that relate CT-based anatomical assessment to standing radiographic pelvic inclination may provide a basis for integrating the present three-dimensional anatomical framework with functional pelvic orientation [20]. Fourth, intraobserver reliability was high for the evaluated morphometric parameters, including the projection-based coverage-area measurements. However, interobserver reliability was not assessed and requires further validation. In addition, the manual cross-grid method used to determine the femoral head center was not directly validated against automated sphere-fitting or volumetric centroid methods. Finally, the cross-sectional design and absence of a pathological comparison group or longitudinal clinical outcomes preclude conclusions regarding diagnostic thresholds, disease progression, or clinical prognosis. Future studies should establish corresponding reference distributions in men to enable evaluation of sex-specific differences and subsequently determine how these reference relationships are altered in dysplastic and osteoarthritic hips. Such studies should also include broader age groups, establish interobserver reliability for the morphometric and projection-based coverage-area measurements, validate the manual femoral head center determination against automated methods, and integrate pelvis-based three-dimensional morphology with functional pelvic and spinopelvic alignment. Within these limitations, the present study provides a population-specific three-dimensional anatomical framework for describing femoral head position, acetabular orientation, and regional coverage within the pelvis.

## Conclusions

This study characterized pelvis-based three-dimensional hip morphology in Japanese women aged 19-59 years without clinically recognized hip disease and demonstrated consistent relationships among femoral head position, acetabular orientation, and regional coverage. In particular, a more anterior femoral head position was associated with greater AV, reduced anterior sector coverage, and greater posterior sector coverage, whereas lateral femoral head position showed weaker associations with these acetabular parameters. The principal pelvis-based positional and angular parameters showed no material evidence of side-to-side or age-related variation within the observed age range, and the major spatial associations were preserved in the sensitivity analyses. These findings provide a population-specific anatomical reference framework for evaluating the spatial relationships among the pelvis, femoral head, and acetabulum beyond conventional coronal measurements alone. Further studies including men, broader age groups, defined hip disorders, and longitudinal outcomes are needed to determine the clinical relevance and generalizability of this framework.
